# Synthesis and Spectroscopic and Luminescent Properties of Er, Yb and Lu Complexes with Cyano-Substituted Phthalocyanine Ligands

**DOI:** 10.3390/molecules27134050

**Published:** 2022-06-23

**Authors:** Dmitrii Erzunov, Ilya Sarvin, Anastasia Belikova, Arthur Vashurin

**Affiliations:** Department of Inorganic Chemistry, Ivanovo State University of Chemistry and Technology, 15300 Ivanovo, Russia; sarvin2002@mail.ru (I.S.); 89051065598@mail.ru (A.B.); vashurin@isuct.ru (A.V.)

**Keywords:** phthalocyanine, rare-earth elements, mono-decker complexes, spectroscopy, fluorescence

## Abstract

Based on 4,4′-[1,3/4-phenilenebis(oxy)]phthalodinitriles, the mixture of phthalocyaninates of various structures with rare-earth metals were obtained by template fusion method minimizing the side polymerization processes. Target monophthalocyaninates were isolated from the reaction mixture and purified using column and then gel permeation chromatography. The compounds were characterized by NMR, IR spectroscopy, mass spectrometry, and elemental analysis. The spectral properties were studied and the aggregation behavior of the synthesized Er, Yb, and Lu phthalocyaninates in chloroform, acetone, and tetrahydrofuran was determined. It has been shown that lutetium complexes with 3,4-dicyanophenoxyphenoxy ligands are the least stable and least resistant to aggregation in solution, while erbium and ytterbium phthalocyaninates proved to be stable in all studied media. The quantum yields and fluorescence lifetimes of the complexes in chloroform and tetrahydrofuran were calculated.

## 1. Introduction

Complexes of rare earth elements (REE) with phthalocyanine derivatives constitute a separate group of compounds due to the specificity of their properties, which is determined by the nature of the central metal atom used. Metal phthalocyaninates are characterized by intense light absorption in the visible region [1,2,3,4], which determines their use as dyes [5,6,7] and elements of optical devices [8,9], while lanthanide complexes, along with the above properties, also exhibit absorption and, accordingly, emission in the near-IR region [10,11]. This opens up prospects for the use of such compounds as optical limiting devices [12,13,14], luminescent films, and sensors [9]. This fact is associated with the presence in the structure of the complex of an atom of a rare earth element, which makes it possible to realize luminescence of the 4f-type [15,16,17].

It is known that complexes of various structures with Er(III), Yb(III), and Lu(III) have high fluorescence quantum yields, which, along with high lifetimes, determine the interest in their study and the prospects for their subsequent application. The structure of the introduced ligand has a significant effect on the luminescent properties exhibited by the complex and can both promote and inhibit the processes of energy transfer between the organic fragment and the complexing atom. Thus, the key task is to search for structures with suitable electronic and steric parameters that are capable of effectively realizing the entire energy potential of the rare earth atom.

It is well known that complexes of phthalocyanines with REE can take both mono-decker and multi-decker forms [18,19]. Sandwich-type complexes of various structures are well studied and attract researchers primarily as potential elements of nonlinear optical devices [20], highly sensitive sensors [21], and electrochromic devices [22]. REE mono-decker phthalocyaninates, in turn, are much less studied due to the lower overall stability of the complexes [23]. However, the available works [23] show that the selection of a suitable peripheral/non-peripheral substitution in the structure of the molecule makes it possible to stabilize the complex in a stable state, which opens up opportunities for studying their properties and their use as semiconductor materials [24] and photo- and electroluminescent applications [25,26]. Moreover, based on stable mono-decker phthalocyaninates, it is possible to obtain various structures of homo- and heteroleptic sandwich structures [27] with the possibility of simply introducing metal ions of various characteristics, which gives the compounds promising properties [28].

One of the key factors limiting the possibility of studying and using phthalocyaninates in the liquid phase is aggregation [29]. In view of the relatively large size of the molecule, this process can be observed even at low concentrations of complexes in solution [30,31,32], which, despite this, often leads to a decrease in the ability of complexes to absorb light [33,34], a decrease in the quantum yields of fluorescence [35] and singlet oxygen [36], and also electrical conductivity [34]. Thus, in order to prevent the occurrence of molecular aggregation, it is necessary to fine-tune the structure of the obtained phthalocyaninates, which consists in the selection of suitable substituents and the central metal ion [37]. It is known that aromatic fragments give the compounds under study good solubility in most commonly used organic solvents [38,39], which opens up the possibility of studying their properties and further application [40,41], on the other hand, the presence of oxygen bridges [42,43] binding aromatic fragments of the substituent, gives these fragments the necessary spatial flexibility, thereby preventing the occurrence of aggregation processes. Thus, we chose the peripheral type of substitution of the phthalocyanine ligand with [3/4-(3,4-dicyanophenoxy)phenoxy fragments, which provides both good solubility and intense properties, for example, spectroscopic [44], catalytic [45], and oxidative-recovery [46]. In the framework of the present work, single-decker complexes of several REE (Er, Yb, Lu) with tetrakis-4-[3/4-(3,4-dicyanophenoxy)phenoxy]-phthalocyanine ligands were obtained and characterized, and the spectroscopic and photophysical properties exhibited by them were studied.

## 2. Results and Discussion

### 2.1. Synthesis of Tetrakis(dicyanophenoxyphenoxy)phthalocyaninates of Rare Earth Elements

**Scheme 1:**
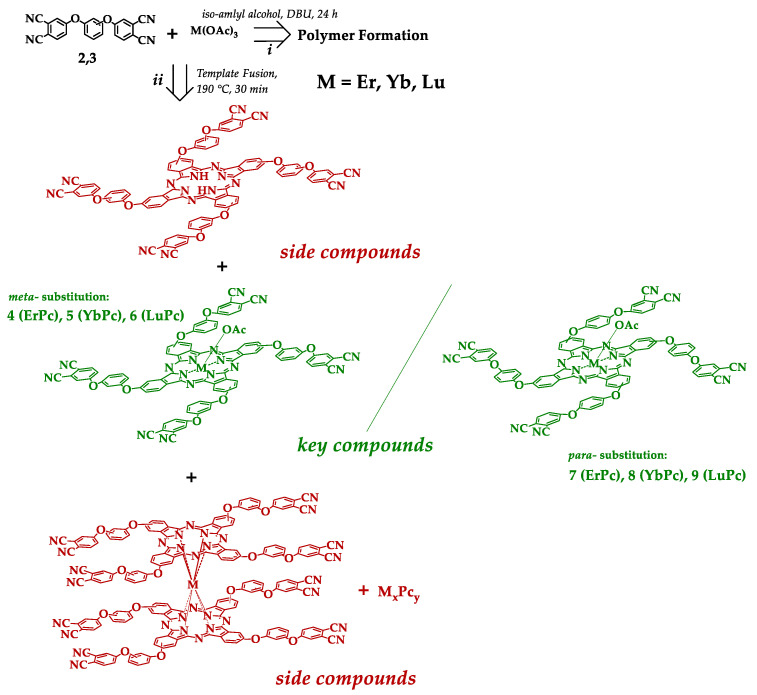
Strategy for obtaining complexes of rare-earth elements with [3/4-(3,4-dicyanophenoxy)phenoxy]-phthalocyanine ligands ((**i**): Reflux in *iso*-amyl alcohol, DBU, 24 h; (**ii**): Template fusion in the absence of solvent, 190 °C, 30 min).

Attempts were made to tetramerize [phenylenebis(oxy)]diphthalonitriles (**2**, **3**) with anhydrous erbium(III), ytterbium(III), and lutetium(III) acetates (Scheme 1i). The reaction was carried out by refluxing in *iso-*amyl alcohol in the presence of 1,8-diazabicyclo[5 .4.0]undec-7-ene (DBU). Nevertheless, as a result, the predominant formation of a polymer alloy of complex composition was observed, and by varying the temperature, the molar ratio of the reactants, and other reaction conditions, it was not possible to increase the yield of the target products (yield < 1%).

This phenomenon is probably due to the presence of a large number of terminal cyano-groups, which, along with their high coordination activity, leads to intense polymerization processes with the formation of a network structure. Thus, in order to obtain the target mono-complexes, it was decided to carry out the reaction under more stringent conditions, namely, by means of template fusion in the absence of a solvent. Previously, we showed [45,46,47] that by varying the ratio of nitrile:metal salt from 4:1 to 8:1, we have a mixture of products of various structures with the predominant formation of mono- (at *v/v* 4:1) or sandwich (at *v/v* 4:1) or sandwich (at *v/v* 8:1) structures, and in the case of the lutetium salt, the inevitable formation of the phthalocyanine ligand is observed. Thus, further, template fusion of the starting phthalonitriles **2** and **3** with anhydrous acetates of rare earth elements was carried out (Scheme 1ii). By varying the reaction conditions, on the whole, it was possible to increase the yields of the complexes by up to ~40% (depending on the structure). The lowest yields were observed in the case of lutetium complexes, which is associated with the smallest radius of the metal, leading to the relative lability of the mono-complex (the occurrence of spontaneous demetallation processes was observed over time).

All compounds obtained in the mass spectra gave the characteristic molecular peaks of the complexes without impurities of the starting reagents or polyphthalocyanine fractions obtained as by-products.

**Figure 1:**
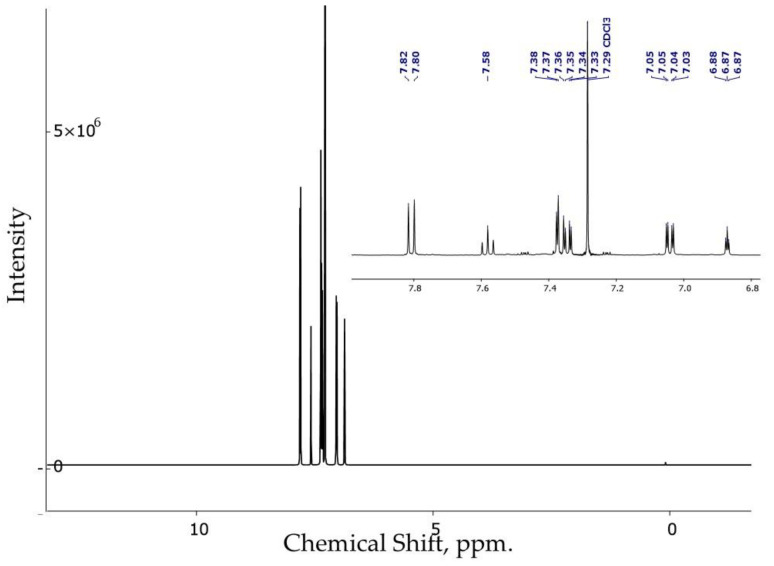
^1^H NMR spectrum of complex **6** in CDCl_3_.

^1^H NMR spectra were obtained for the compounds (Figure 1). The paramagnetic nature of the central metal atoms included in the structures of the obtained complexes greatly complicates the analysis and interpretation of the obtained spectral patterns, causing specific broadening of signals and their shifts [48], however, we have shown earlier [46] that for sandwich type complexes, because of the relative remoteness of the periphery from the complexing agent, there are no significant shifts in the proton signals of peripheral substituents in the ^1^H NMR spectra of the obtained phthalocyaninates, while the proton signals of macrocyclic fragments are shifted to the upfield.

A similar picture is also observed for mono-phthalocyaninates, which is probably due to the release of the rare-earth metal atom directly from the coordination cavity of the compound. However, for lutetium diamagnetic complexes **6** and **9**, well-resolved spectra on the ^1^H nucleus were obtained, on the basis of which, in combination with the data of other research and instrumental methods of analysis, we can conclude that the synthesis strategy was successfully chosen to obtain the given rare-earth phthalocyaninates and the uniqueness of the definite structure of the complexes.

### 2.2. Spectroscopic and Aggregation Properties of Complexes in Organic Media

The spectral properties of the obtained compounds were studied, the light absorption maxima in the visible region were determined in a number of organic solvents, and the extinction coefficients were determined. However, the use of compounds in the liquid phase has a number of limitations associated with the occurrence of such side processes as aggregation, which can adversely affect all exhibited properties [49,50]. Thus, the aggregation properties of the complexes were studied, and the concentration ranges of the aggregation stability of the compounds in the studied media were determined.

**Figure 2:**
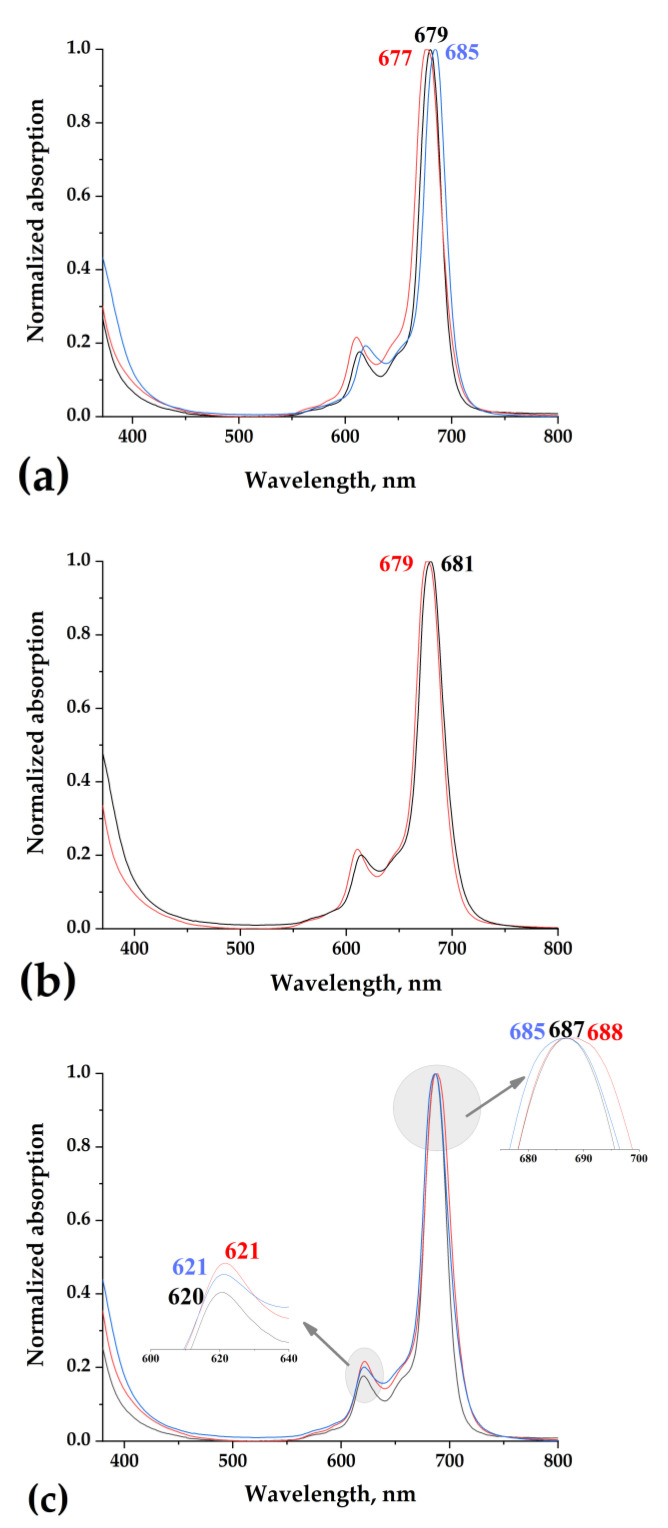
(**a**): Normalized electronic absorption spectra of compound **4** in acetone (red line), tetrahydrofuran (black line), and chloroform (blue); (**b**): Normalized electronic absorption spectra for compounds **4** (red line) and **7** (black line) in tetrahydrofuran; (**c**): Normalized electronic absorption spectra for compounds **4**, **5,** and **6** in chloroform.

Figure 2a shows the electronic absorption spectra of the compounds in chloroform, acetone, and tetrahydrofuran. In the case of chloroform, the largest bathochromic shift of the absorption maxima is observed for all the studied complexes, which can be caused by a change in polarity in the series acetone-tetrahydrofuran-chloroform, and, at the same time, with a low coordination ability of chloroform, leading to less solvation of the coordination center of molecules and, as a result, greater accessibility. Thus, passing from acetone to tetrahydrofuran, the greatest contribution to the red-wave shift corresponds to the solvatochromic effect, while when passing to chloroform, in addition to solvatochromism, one should also take into account the degree of solvation of the coordination center of the molecule. Moreover, a slight bathochromic shift characterizes the transition from complexes with meta-substitution in the central ring of the peripheral substituent to para-analogs in all studied media (Figure 2b).

In turn, in the series Er-Yb-Lu of the coordination centers of the complexes, a regular bathochromic shift (Table 1, Figure 2c) of the absorption spectra in chloroform, acetone, and tetrahydrofuran is observed, which is explained by a decrease in the atomic radius in the described series, resulting in a change in orbital energy.

**Figure 3:**
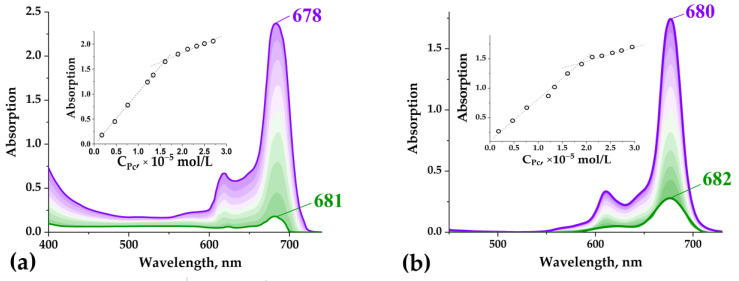
Changes in the electronic absorption spectra for solutions of compounds **6** (**a**) and **9** (**b**) in acetone upon dilution. Inserts show the dependencies of the optical density on the concentration of phthalocyanine in the solution.

For solutions of compounds **4**–**9** in all studied media, aggregation characteristics were determined. Based on the constructed plots of the dependence of the change in optical density on the concentration of phthalocyanine upon dilution, it was shown that the introduction of 3/4-(3,4-dicyanophenoxy)phenoxy- fragments into the peripheral positions of the phthalocyanine macroring contributes to the resistance of the complexes to aggregation in chloroform and tetrahydrofuran media. For lutetium complexes in acetone (**6**, **9**), deviations of the dependence from a linear form were observed at concentrations of 1.52 × 10^−5^ and 1.77 × 10^−5^ mol/L, respectively, along with a bathochromic shift of the electronic absorption spectra, which may indirectly indicate aggregation equilibria (Figure 3). The concentration ranges below the indicated values are represented by straight-line dependences, which, together with the nature of the change in the spectral pattern in both cases, gives grounds to judge the existence of compounds in an aggregated form at concentrations exceeding certain values in an acetone medium, and in a monomeric form at lower values.

In addition, one of the pieces of evidence for the occurrence of side processes in solutions of compounds **6** and **9** is the obtained values of the total width at half-height of the absorption maximum (Table S1). With sequential dilution of solutions of these complexes, a regular decrease in Δλ was observed to critical points corresponding to the concentrations of macrocycles in the solution presented above.

Thus, the obtained erbium and ytterbium phthalocyanines are presented in solution in monomeric form even at high concentrations (up to 6 × 10^−5^ mol/L) in all the studied media, lutetium complexes showed good aggregation stability in chloroform and tetrahydrofuran media and limited in the case of acetone, which may be due to the specifics of the electronic structure of the lutetium atom, namely the highest electronegativity and, at the same time, the smallest atomic radius in the presented series of metals.

In comparison with the complexes of the described ligands with *d*-metals [45], a regular bathochromic shift of the electronic absorption spectra by 20–30 nm, depending on the structure, is observed, which is associated with the introduction of heavy atoms of rare earth elements. The values of the light absorption coefficients remain practically unchanged (the difference is less than 2.5% in the chloroform medium). In the transition to sandwich bisphthalocyaninates, the opposite picture is observed—the ability of molecules to absorb light increases (up to 12%), and at the same time, the absorption maxima change only by 2–3 nm.

### 2.3. Photophysical Properties of Complexes in Organic Media

For solutions of compounds **4**–**9**, photophysical characteristics were calculated in chloroform, acetone, and tetrahydrofuran media (Table 2).

It was found that, upon going from meta-substitution in the central ring of the peripheral substituent to the para-analog, a regular decrease in the fluorescence quantum yields by 5–10% is observed, which is probably associated with the greater steric flexibility of the para-substituted fragments. In addition, in the Er-Yb-Lu series, the fluorescence quantum yields of the complexes decrease due to the manifestation of the heavy atom effect.

In the studied series of solvents chloroform-acetone-tetrahydrofuran, a regular decrease in the fluorescence quantum yields of the studied rare earth phthalocyaninates is observed, which is associated with an increase in the polarity of the medium, on the one hand, and an increase in the coordination strength of the solvent, leading to greater solvation of the coordination center of the macroheterocyclic fragment, on the other.

Deviations from certain regularities were found for compounds **6** and **9** in acetone, which correlates with the data of the spectroscopic studies performed and is explained by the aggregation of molecules in solution, leading to fluorescence quenching (Table 2).

**Figure 4:**
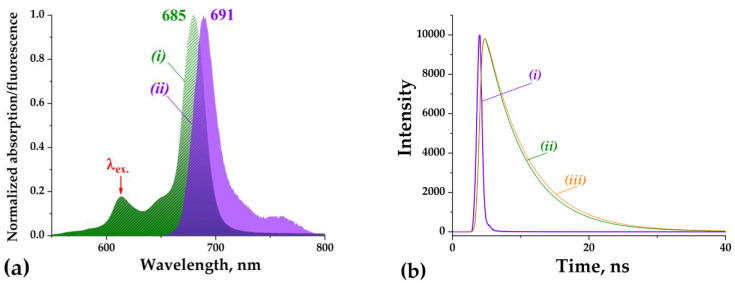
(**a**): Electron absorption (*i*) and fluorescence (*ii*) spectra for compound **4** in chloroform; (**b**): Fluorescence decay curves for compounds **4** (*ii*) and **9** (*iii*) in chloroform and LUDOX solution in water (standard) (*i*).

The fluorescence lifetime is the average time during which a molecule is in an excited state before a direct transition to the ground state. The τ_f_ values were calculated using the Strickler–Berg equation using the time-correlated photon counting (TCSPC) method, the essence of which is to detect single photons emitted when a molecule is excited by periodic light pulses. In the study, the decay curves of all studied complexes of rare earth elements were described by a monoexponential function (Figure 4b). It is shown that the spatial factor, namely the type of substitution in the central ring of the peripheral substituent of the studied molecules, has practically no effect (within 1.7%) on the fluorescence lifetime of the compounds. In turn, the change of the solvent from chloroform to acetone and then tetrahydrofuran is accompanied by a successive decrease in the fluorescence lifetime, which is apparently explained by an increase in the solvation of the molecule upon replacement of the solvent in this series.

## 3. Materials and Methods

### 3.1. Reagents and Equipment

Electronic absorption spectra in the spectral range 190–1100 nm were recorded using UNICO2800 (United Productsand Instruments, Dayton, NJ, USA) and AvaSpec-ULS2048CL-EVO (Avantes, Louisville, KY, USA) spectrophotometers in chloroform, tetrahydrofuran, and acetone using quartz cuvettes with an optical path length of 1 cm. Fluorescent properties were studied on a Cary Eclipse Varian-Agilent instrument (Agilent, Santa Clara, CA, USA). Fluorescence lifetimes were determined using a FluoTime 300 (PicoQuant, Berlin, Germany) fluorescence spectrometer. Elemental analysis of the percentage of hydrogen, carbon, nitrogen, and oxygen atoms was performed on a CHNS-OFlashEA, 1112 analyzer (Thermo Quest, Milan, Italy). Time-of-flight matrix-activated laser ionization (MALDI mass analysis) was performed on an Axima Confidence Time-of-Flight Mass-Spectrometer (Shimadzu, London, UK) by preparing concentrated (about 10^−3^ mol/L) solutions of the complexes in tetrahydrofuran, applying them to a matrix, and subsequent ionization. α-cyanohydroxycinnamic acid was used as a matrix. IR spectra were recorded in the range 450–4500 cm^−1^ on an IRAffinity-1S spectrometer (Shimadzu, Kyoto, Japan) from dry ground powders of compounds without additional sample preparation.

Purification of the complexes was carried out using gradient column chromatography with silica gel as a sorbent (200–400 mesh), as well as gel permeation chromatography on Bio-Beads S-X1 beads (Bio-Rad, Hercules, CA, USA). The progress of the reactions and the success of the purification were checked by thin layer chromatography.

Acetone, chloroform, tetrahydrofuran (THF), lutetium(III) acetate, erbium(III) acetate, ytterbium(III) acetate, *iso*-amyl alcohol, ethyl alcohol (Sigma-Aldrich) were used without extra purification.

4,4′-[1,3-phenylenebis(oxy)]phthalonitrile (**2**) and 4,4′-[1,4-phenylenebis(oxy)]phthalodinitrile (**3**) were obtained by nucleophilic substitution in 4-nitrophthalonitrile (**1**) and characterized according to [44].

### 3.2. Synthetic Part

#### General Route for Obtaining Complexes of Mono- Phthalocyaninates of Rare Earth Elements

4,4′-[1,3/4-Phenylenebis(oxy)]phthalodinitrile (**2**, **3**) and anhydrous acetate of the corresponding metal (ErAc_3_, YbAc_3_, LuAc_3_) were mixed in a ceramic crucible and heated to 190°C for 30 min to complete transition of the reaction mass into the liquid phase. After cooling, the product was successively washed on a Schott filter with ethanol, an aqueous alkaline solution, and water, dried, and then washed off the filter with tetrahydrofuran. Compounds were purified by column (SiO_2_, CHCl_3_-THF gradient) and gel permeation (Bio-Beads S-X1, 2.5% ethanol in CHCl_3_) chromatography, each step was controlled by thin layer chromatography.

Acetate tetrakis-4-[3-(3,4-dicyanophenoxy)phenoxy]phthalocyaninato erbium (**4**)

Yield: 40%. IR, ν_max_, cm^−1^ 3057, 2917, 2851 (C-H), 2235 (C≡N), 1594, 1483 (C=C), 1252 (Ar-O-Ar). ^1^H-NMR (500 MHz, CDCl_3_): δ, ppm. 7.57–6.48 (m), 5.01 (m), 3.00–1.56 (m). MS (MALDI-TOF): *m/z* 1675.70 [M]+, calcd. 1675.69. Calculated for C_90_H_43_N_16_O_10_Er: C 64.51, H 2.59, N 13.37, O 9.55; found: C 64.50, H 2.58, N 13.37, O 9.57.

Acetate tetrakis-4-[3-(3,4-dicyanophenoxy)phenoxy]phthalocyaninato ytterbium (**5**)

Yield: 37%. IR, ν_max_, cm^−1^ 3059, 2913, 2857 (C-H), 2233 (C≡N), 1591, 1484 (C=C), 1247 (Ar-O-Ar). ^1^H-NMR (500 MHz, CDCl_3_): δ, ppm. 7.61–6.39 (m), 5.18 (m), 3.11–1.62 (m). MS (MALDI-TOF): *m/z* 1681.49 [M]+, calcd. 1681.49. Calculated for C_90_H_43_N_16_O_10_Yb: C 64.29, H 2.58, N 13.33, O 9.51; found: C 64.28, H 2.58, N 13.32, O 9.53.

Acetate tetrakis-4-[3-(3,4-dicyanophenoxy)phenoxy]phthalocyaninato lutetium (**6**)

Yield: 25%. IR, ν_max_, cm^−1^ 3055, 2916, 2850 (C-H), 2231 (C≡N), 1591, 1486 (C=C), 1252 (Ar-O-Ar). ^1^H-NMR (500 MHz, CDCl3): δ, ppm. 7.82 (s), 7.80 (s), 7.58 (t), 6.87 (t), 7.37–7.36 (dd), 7.35–7.33 (dd), 7.05–7.03 (dd). MS (MALDI-TOF): *m/z* 1683.40 [M]+, calcd. 1683.39. Calculated for C_90_H_43_N_16_O_10_Lu: C 64.21, H 2.57, N 13.31, O 9.50; found: C 64.21, H 2.56, N 13.30, O 9.52.

Acetate tetrakis-4-[4-(3,4-dicyanophenoxy)phenoxy]phthalocyaninato erbium (**7**)

Yield: 36%. IR, ν_max_, cm^−1^ 3058, 2916, 2849 (C-H), 2230 (C≡N), 1595, 1483 (C=C), 1249 (Ar-O-Ar). ^1^H-NMR (500 MHz, CDCl_3_): δ, ppm. 7.55–6.39 (m), 5.21 (m), 3.16 -1.57 (m). MS (MALDI-TOF): *m/z* 1675.70 [M]+, calcd. 1675.69. Calculated for C_90_H_43_N_16_O_10_Er: C 64.51, H 2.59, N 13.37, O 9.55; found: C 64.50, H 2.57, N 13.37, O 8.52.

Acetate tetrakis-4-[4-(3,4-dicyanophenoxy)phenoxy]phthalocyaninato ytterbium (**8**) 

Yield: 33%. IR, ν_max_, cm^−1^ 3060, 2915, 2848 (C-H), 2233 (C≡N), 1594, 1480 (C=C), 1248 (Ar-O-Ar). ^1^H-NMR (500 MHz, CDCl_3_): δ, ppm. 7.63–6.52 (m), 5.32 (m), 3.16–1.67 (m). MS (MALDI-TOF): *m/z* 1681.49 [M]+, calcd. 1681.49. Calculated for C_90_H_43_N_16_O_10_Yb: C 64.29, H 2.58, N 13.33, O 9.51; found: C 64.30, H 2.58, N 13.32, O 9.51.

Acetate tetrakis-4-[4-(3,4-dicyanophenoxy)phenoxy]phthalocyaninato lutetium (**9**)

Yield: 20%. IR, ν_max_, cm^−1^ 3057, 2912, 2852 (C-H), 2229 (C≡N), 1560, 1486 (C=C), 1254 (Ar-O-Ar). ^1^H-NMR (500 MHz, CDCl_3_): δ, ppm. 7.83 (s), 7.81 (s), 7.60 (t), 6.88 (t), 7.38–7.37 (dd), 7.36–7.34 (dd), 7.07–7.05 (dd). MS (MALDI-TOF): *m/z* 1683.40 [M]+, calcd. 1683.40. Calculated for C_90_H_43_N_16_O_10_Lu: C 64.21, H 2.57, N 13.31, O 9.50; found: C 64.20, H 2.57, N 13.31, O 9.49.

### 3.3. Study of Spectroscopic and Aggregation Properties

The spectroscopic properties were studied by the spectrophotometric method, recording spectra in the visible range. Electronic absorption spectra of compound solutions were recorded in chloroform, acetone, and tetrahydrofuran in the concentration range from 5 × 10^−6^ to 5 × 10^−5^ M.

### 3.4. Study of Fluorescent Properties

The emission spectra were recorded for solutions of phthalocyaninates in chloroform, acetone, and tetrahydrofuran upon excitation at the absorption maximum of the vibrational satellite. The optical absorption density of solutions at the excitation wavelength did not exceed 0.1 to avoid concentration quenching of fluorescence. Unsubstituted zinc phthalocyaninate in pyridine (Φ_fluor_ = 0.3) was used as a standard [47]. Fluorescence lifetimes were calculated using the EasyTau 2 software package (version 2.2.3293, PicoQuant, Berlin, Germany) using LUDOX in water as a standard.

The fluorescence quantum yields of the complexes were calculated using the formula:

(1) $$\Phi_{x}=\frac{A_{s}F_{x}n_{x}^{2}}{A_{x}F_{s}n_{s}^{2}}\Phi_{s}$$

where Φ*_x_* and Φ*_s_* are the fluorescent quantum yields of the compound and standard, respectively, *A_s_* and *A_x_* are the optical density values at the maximum fluorescence excitation for the compound and standard, respectively, *F_x_* and *F_s_* are the area under the graph of the obtained fluorescence spectra for the compound and standard, respectively, *n_x_* and n_s_ are the refractive indices of the solvent for the studied solutions of the compound and standard, respectively.

## 4. Conclusions

Novel complexes of erbium(III), ytterbium(III), and lutetium(III) with [3/4-(3,4-dicyanophenoxy)phenoxy]phthalocyanine ligands were obtained and characterized. The template fusion method in the absence of a solvent has proven to be the most efficient approach in obtaining compounds prone to polymerization. Lutetium compounds showed the lowest yields as a result of the synthesis due to the smallest atomic radius of the metal. It has been shown that 3/4-(3,4-dicyanophenoxy)phenoxy- substitution, along with good solubility in organic media, imparts aggregation stability to molecules (limited in the case of lutetium complexes in acetone) in them, which made it possible to study the spectral properties in chloroform media, acetone and tetrahydrofuran—the values of absorption maxima in the visible region were determined, the molar coefficients of light absorption were determined. It was found that such a substitution leads to an increase in the fluorescence quantum yields of the complexes and their lifetime, which is uncharacteristic for monophthalocyaninates of rare earth elements, which, together with high values of molar light absorption coefficients, opens up prospects for the use of compounds as, for example, promising dyes/fluorescent dyes or biomarkers. It has been determined that the transition from complexes with meta-substitution in the central ring of the peripheral substituent to para- is accompanied by a regular bathochromic shift of the electronic absorption spectra in all studied media and also leads to a decrease (up to 10%) in fluorescence quantum yields, however, the their fluorescence lifetime values remain virtually unchanged. 

## Figures and Tables

**Table 1:** Spectroscopic characteristics of compounds **4**–**9** in organic media.

Compound	Qmax, nm (lgε)		
	CHCl3	Acetone	THF
**4**	685 (4.34)	677 (4.29)	679 (4.33)
**5**	687 (4.89)	677 (4.33)	680 (4.41)
**6**	688 (4.32)	678 (4.27)	682 (4.28)
**7**	687 (4.40)	678 (4.30)	681 (4.37)
**8**	689 (4.29)	679 (4.26)	682 (4.25)
**9**	690 (4.38)	680 (4.30)	682 (4.33)

**Table 2:** Photophysical characteristics of compounds **4**–**9** in organic media.

Compound	CHCl_3_		Acetone		THF	
	Φ_x_	τ_f_, [ns]	Φ_x_	τ_f_, [ns]	Φ_x_	τ_f_, [ns]
**4**	0.19	6.01	0.17	5.92	0.15	5.79
**5**	0.18	5.93	0.15	5.78	0.12	5.66
**6**	0.09	5.84	0.08	5.71	0.06	5.60
**7**	0.17	6.00	0.16	5.90	0.13	5.79
**8**	0.17	5.93	0.15	5.77	0.11	5.67
**9**	0.06	5.84	0.06	5.71	0.04	5.58

## Data Availability

Not applicable.

## Supplementary Materials

The following supporting information can be downloaded at https://www.mdpi.com/article/10.3390/molecules27134050/s1. Figure S1. Electronic absorption spectrum of tetrakis-4-[3-(3,4-dicyanophenoxy)phenoxy]phthalocyaninato erbium acetate (**4**) in chloroform; Figure S2. Electronic absorption spectrum of ytterbium tetrakis-4-[3-(3,4-dicyanophenoxy)phenoxy]-phthalocyaninato acetate (**5**) in chloroform; Figure S3. Electronic absorption spectrum of lutetium tetrakis-4-[3-(3,4-dicyanophenoxy)phenoxy]-phthalocyaninato acetate (**6**) in chloroform; Figure S4. Electronic absorption spectrum of tetrakis-4-[3-(3,4-dicyanophenoxy)phenoxy]-phthalocyaninato erbium acetate (**4**) in acetone; Figure S5. Electronic absorption spectrum of ytterbium tetrakis-4-[3-(3,4-dicyanophenoxy)phenoxy]-phthalocyaninato acetate (**5**) in acetone; Figure S6. Electronic absorption spectrum of lutetium tetrakis-4-[3-(3,4-dicyanophenoxy)phenoxy]-phthalocyaninato acetate (**6**) in acetone; Figure S7. Electronic absorption spectrum of tetrakis-4-[3-(3,4-dicyanophenoxy)phenoxy]-phthalocyaninato erbium acetate (**4**) in tetrahydrofuran; Figure S8. Electronic absorption spectrum of tetrakis-4-[3-(3,4-dicyanophenoxy)phenoxy]-phthalocyaninato ytterbium acetate (**5**) in tetrahydrofuran; Figure S9. Electronic absorption spectrum of lutetium tetrakis-4-[3-(3,4-dicyanophenoxy)phenoxy]-phthalocyaninato acetate (**6**) in tetrahydrofuran; Figure S10. Electronic absorption spectrum of tetrakis-4-[4-(3,4-dicyanophenoxy)phenoxy]-phthalocyaninato erbium acetate (**7**) in chloroform; Figure S11. Electronic absorption spectrum of ytterbium tetrakis-4-[4-(3,4-dicyanophenoxy)phenoxy]-phthalocyaninato acetate (**8**) in chloroform; Figure S12. Electronic absorption spectrum of lutetium tetrakis-4-[4-(3,4-dicyanophenoxy)phenoxy]phthalocyaninato acetate (**9**) in chloroform; Figure S13. Electronic absorption spectrum of tetrakis-4-[4-(3,4-dicyanophenoxy)phenoxy]-phthalocyaninato erbium acetate (**7**) in acetone; Figure S14. Electronic absorption spectrum of ytterbium tetrakis-4-[4-(3,4-dicyanophenoxy)phenoxy]-phthalocyaninato acetate (**8**) in acetone; Figure S15. Electronic absorption spectrum of lutetium tetrakis-4-[4-(3,4-dicyanophenoxy)phenoxy]phthalocyaninato acetate (**9**) in acetone; Figure S16. Electronic absorption spectrum of tetrakis-4-[4-(3,4-dicyanophenoxy)phenoxy]-phthalocyaninato erbium acetate (**7**) in tetrahydrofuran; Figure S17. Electronic absorption spectrum of tetrakis-4-[4-(3,4-dicyanophenoxy)phenoxy]-phthalocyaninato ytterbium acetate (**8**) in tetrahydrofuran; Figure S18. Electronic absorption spectrum of lutetium tetrakis-4-[4-(3,4-dicyanophenoxy)phenoxy]phthalocyaninato acetate (**9**) in tetrahydrofuran; Figure S19. The structural formula of Acetate tetrakis-4-[3-(3,4-dicyanophenoxy)phenoxy]phthalocyaninato erbium (**4**); Figure S20. The structural formula of Acetate tetrakis-4-[3-(3,4-dicyanophenoxy)phenoxy]phthalocyaninato ytterbium (**5**); Figure S21. The structural formula of Acetate tetrakis-4-[3-(3,4-dicyanophenoxy)phenoxy]phthalocyaninato lutetium (**6**); Figure S22. The structural formula of Acetate tetrakis-4-[4-(3,4-dicyanophenoxy)phenoxy]phthalocyaninato erbium (**7**); Figure S23. The structural formula of Acetate tetrakis-4-[4-(3,4-dicyanophenoxy)phenoxy]phthalocyaninato ytterbium (**8**); Figure S24. The structural formula of Acetate tetrakis-4-[4-(3,4-dicyanophenoxy)phenoxy]phthalocyaninato lutetium (**9**); Figure S25. ^1^H NMR spectrum of Acetate tetrakis-4-[3-(3,4-dicyanophenoxy)phenoxy]phthalocyaninato erbium (**4**) in CDCl_3_; Figure S26. ^1^H NMR spectrum of Acetate tetrakis-4-[3-(3,4-dicyanophenoxy)phenoxy]phthalocyaninato ytterbium (**5**) in CDCl_3_; Figure S27. ^1^H NMR spectrum of Acetate tetrakis-4-[4-(3,4-dicyanophenoxy)phenoxy]phthalocyaninato erbium (**7**) in CDCl_3_; Figure S28. ^1^H NMR spectrum of Acetate tetrakis-4-[4-(3,4-dicyanophenoxy)phenoxy]phthalocyaninato ytterbium (**8**) in CDCl_3_; Figure S29. ^1^H NMR spectrum of Acetate tetrakis-4-[4-(3,4-dicyanophenoxy)phenoxy]phthalocyaninato lutetium (**9**) in CDCl_3_. Table S1. FWHT determinations for compounds **4**–**9** in organic media.
