# Supplementary material for: Synthesis and Spectroscopic and Luminescent Properties of Er, Yb and Lu Complexes with Cyano-Substituted Phthalocyanine Ligands
Source: Molecules. 2022 Jun 23;27(13):4050. doi: 10.3390/molecules27134050 (PMC9267990; doi:10.3390/molecules27134050)
Supplement: Supplementary file 1 [file molecules-27-04050-s001.zip › molecules-1757940-supplementary.pdf]

Supplementary Material

Synthesis and Spectroscopic and Luminescent Properties of Er, Yb and Lu Complexes with Cyano-Substituted Phthalocyanine Ligands

Dmitrii Erzunov *, Ilya Sarvin, Anastasia Belikova and Arthur Vashurin

Department of Inorganic Chemistry, Ivanovo State University of Chemistry and Technology, Sheremetevsky ave.,7, 15300, Ivanovo, Russia; sarvin2002@mail.ru (I.S.); 89051065598@mail.ru (A.B.); vashurin@isuct.ru (A.V.)

* Correspondence: demon_erz@mail.ru

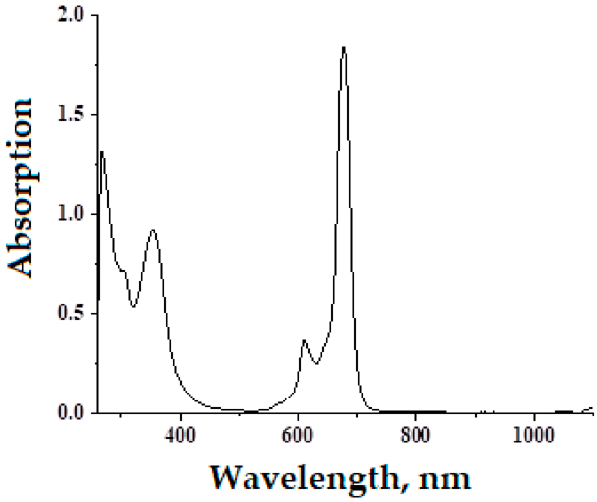

Figure S1. Electronic absorption spectrum of tetrakis-4-[3-(3,4-dicyanophenoxy)phenoxy]phthalocyaninato erbium acetate (**4**) in chloroform;

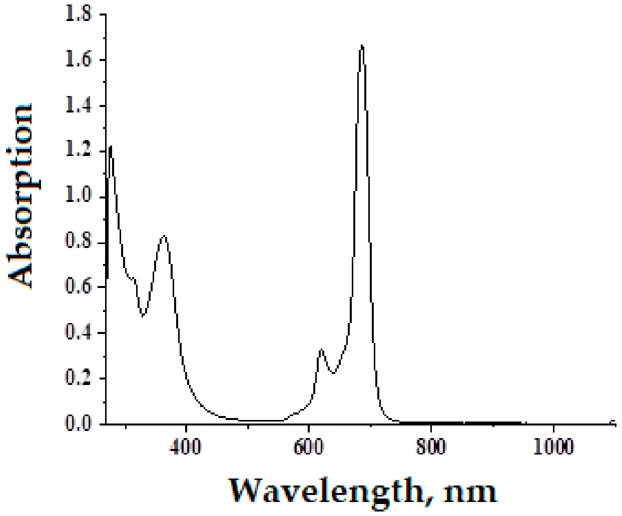

Figure S2. Electronic absorption spectrum of ytterbium tetrakis-4-[3-(3,4-dicyanophenoxy)phenoxy]-phthalocyaninato acetate (**5**) in chloroform;

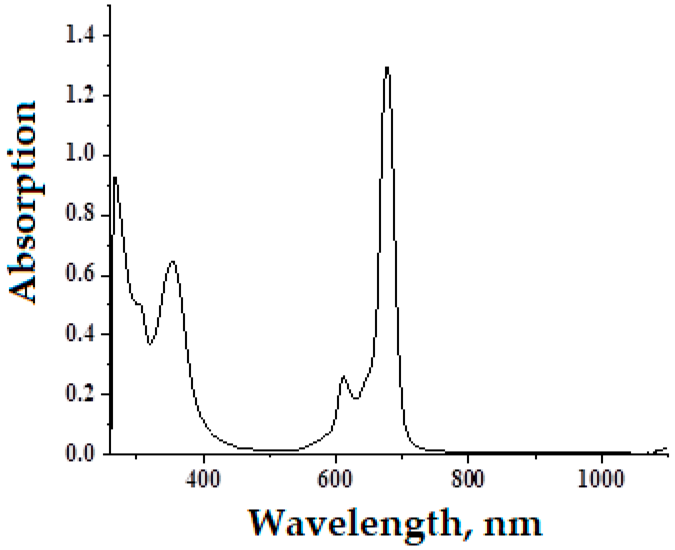

Figure S3. Electronic absorption spectrum of lutetium tetrakis-4-[3-(3,4-dicyanophenoxy)phenoxy]-phthalocyaninato acetate (**6**) in chloroform;

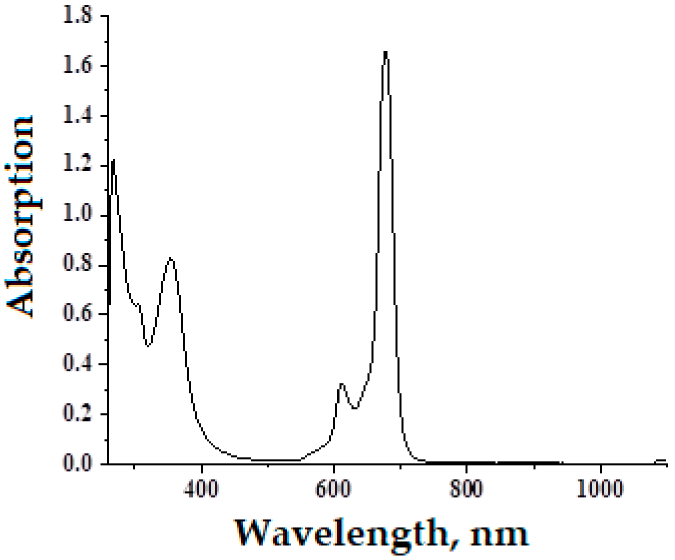

Figure S4. Electronic absorption spectrum of tetrakis-4-[3-(3,4-dicyanophenoxy)phenoxy]-phthalocyaninato erbium acetate (**4**) in acetone;

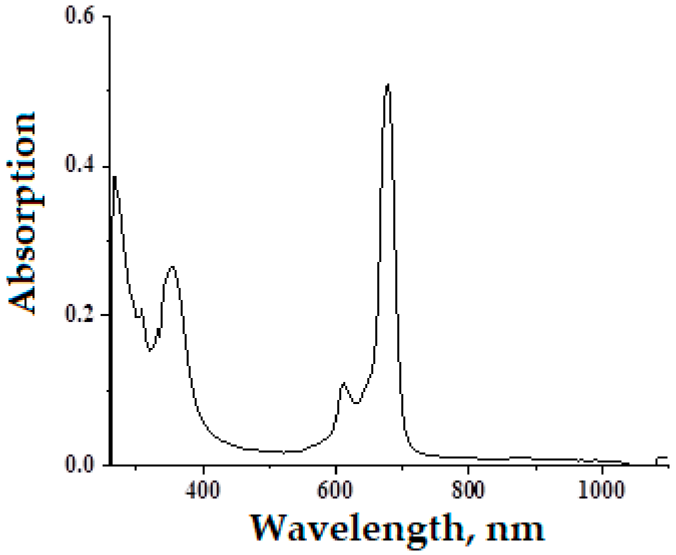

Figure S5. Electronic absorption spectrum of ytterbium tetrakis-4-[3-(3,4-dicyanophenoxy)phenoxy]-phthalocyaninato acetate (**5**) in acetone;

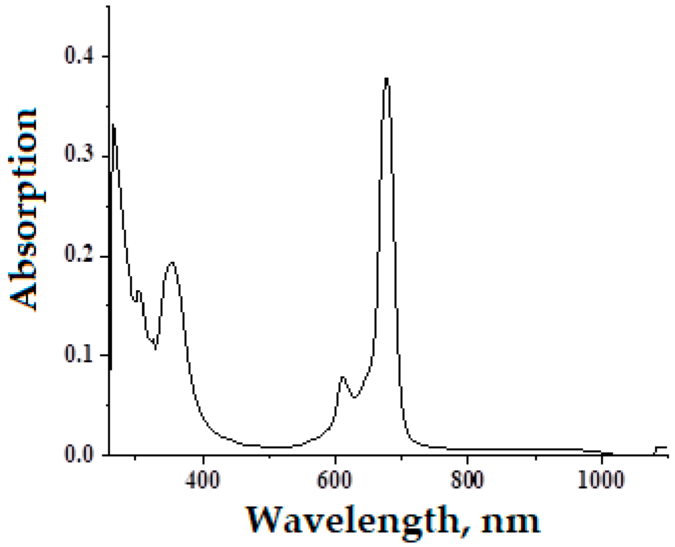

Figure S6. Electronic absorption spectrum of lutetium tetrakis-4-[3-(3,4-dicyanophenoxy)phenoxy]-phthalocyaninato acetate (**6**) in acetone;

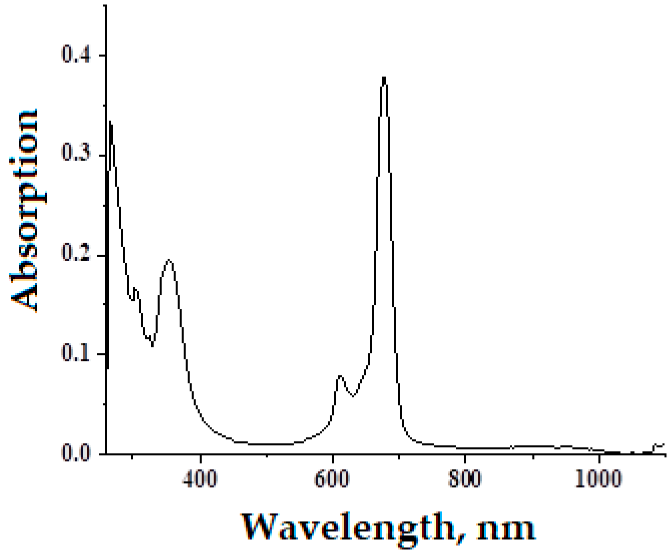

Figure S7. Electronic absorption spectrum of tetrakis-4-[3-(3,4-dicyanophenoxy)phenoxy]-phthalocyaninato erbium acetate (**4**) in tetrahydrofuran;

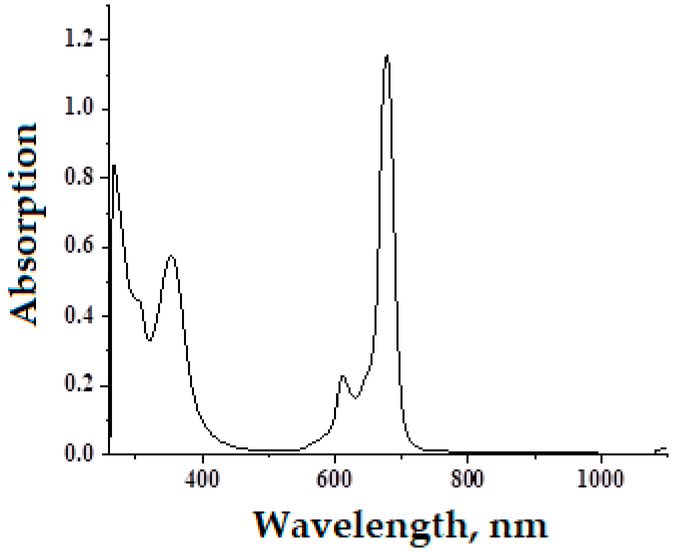

Figure S8. Electronic absorption spectrum of tetrakis-4-[3-(3,4-dicyanophenoxy)phenoxy]-phthalocyaninato ytterbium acetate (**5**) in tetrahydrofuran;

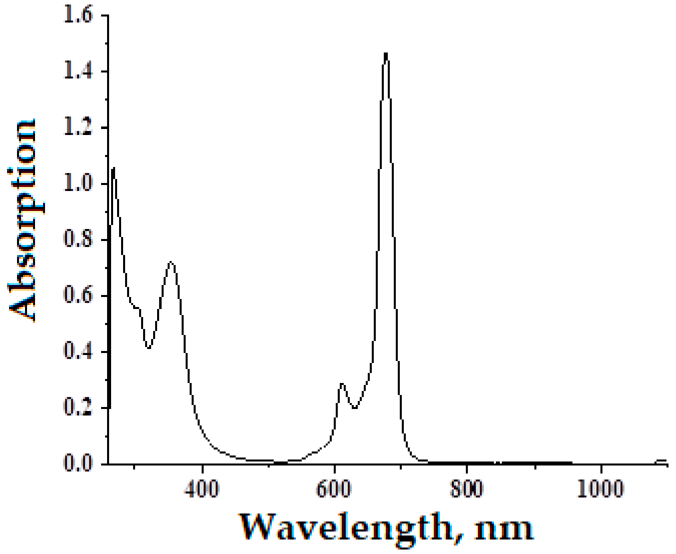

Figure S9. Electronic absorption spectrum of lutetium tetrakis-4-[3-(3,4-dicyanophenoxy)phenoxy]-phthalocyaninato acetate (**6**) in tetrahydrofuran;

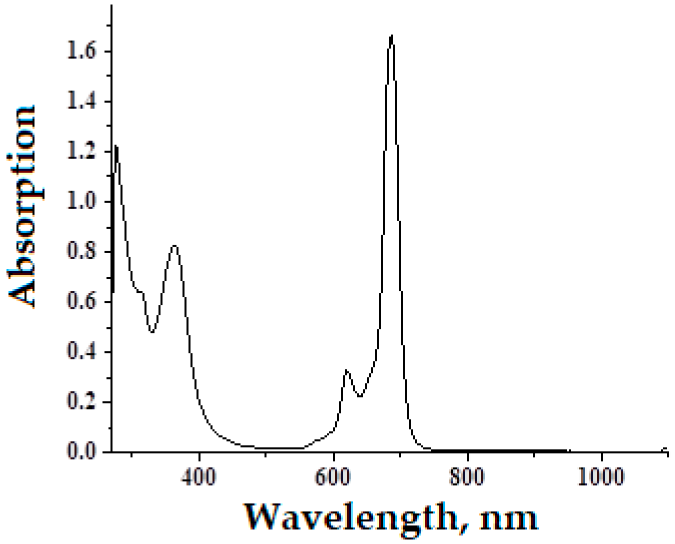

Figure S10. Electronic absorption spectrum of tetrakis-4-[4-(3,4-dicyanophenoxy)phenoxy]-phthalocyaninato erbium acetate (**7**) in chloroform;

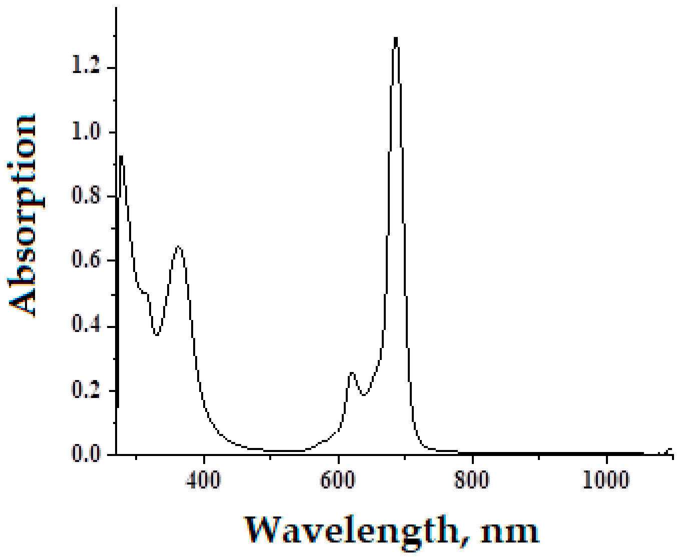

Figure S11. Electronic absorption spectrum of ytterbium tetrakis-4-[4-(3,4-dicyanophenoxy)phenoxy]-phthalocyaninato acetate (**8**) in chloroform;

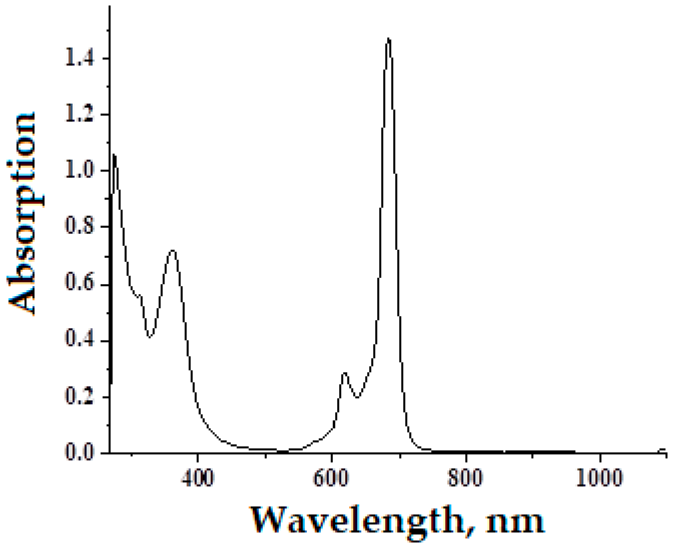

Figure S12. Electronic absorption spectrum of lutetium tetrakis-4-[4-(3,4-dicyanophenoxy)phenoxy]phthalocyaninato acetate (**9**) in chloroform;

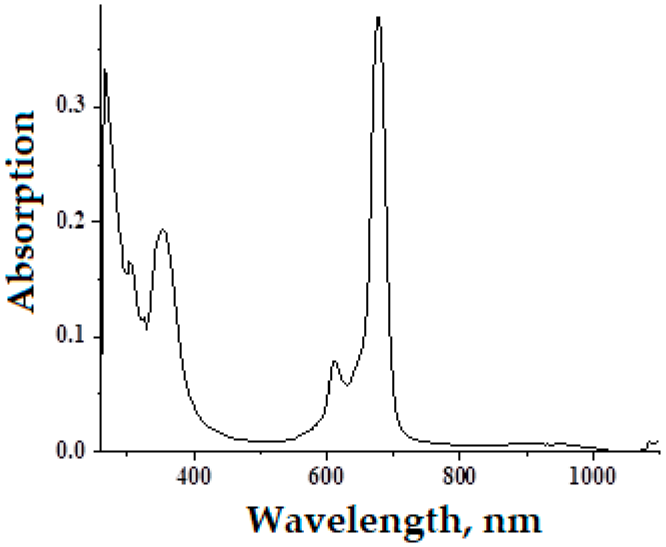

Figure S13. Electronic absorption spectrum of tetrakis-4-[4-(3,4-dicyanophenoxy)phenoxy]-phthalocyaninato erbium acetate (**7**) in acetone;

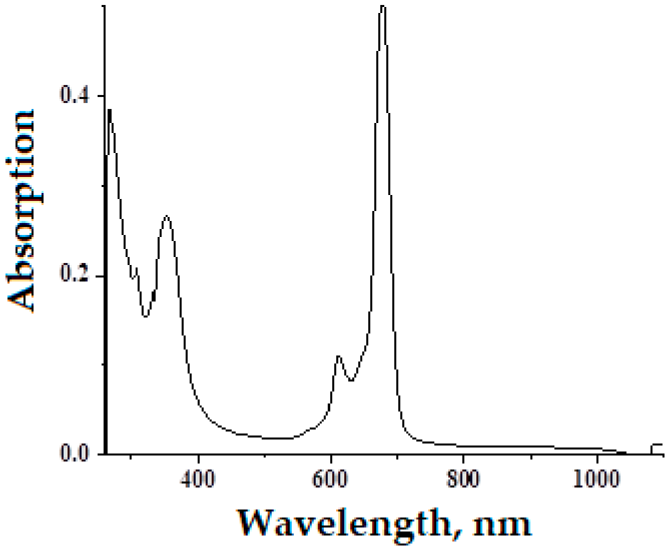

Figure S14. Electronic absorption spectrum of ytterbium tetrakis-4-[4-(3,4-dicyanophenoxy)phenoxy]-phthalocyaninato acetate (**8**) in acetone;

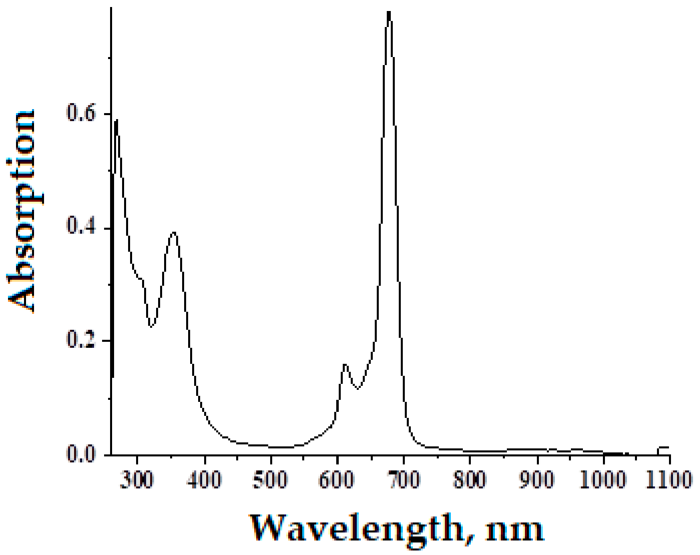

Figure S15. Electronic absorption spectrum of lutetium tetrakis-4-[4-(3,4-dicyanophenoxy)phenoxy]phthalocyaninato acetate (**9**) in acetone;

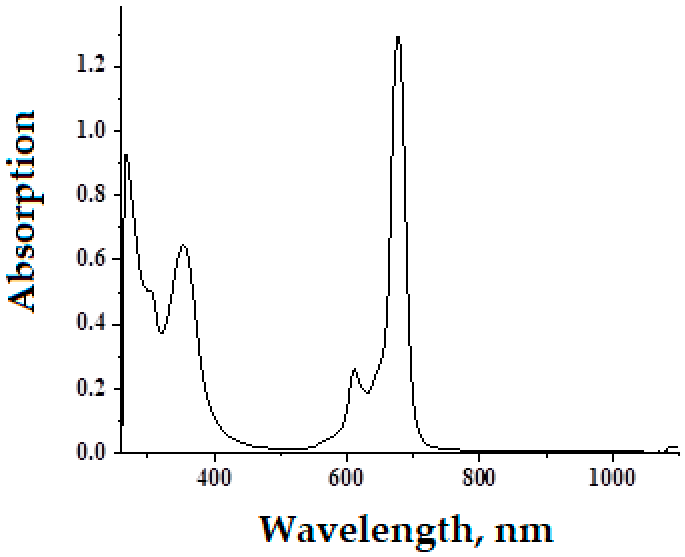

Figure S16. Electronic absorption spectrum of tetrakis-4-[4-(3,4-dicyanophenoxy)phenoxy]-phthalocyaninato erbium acetate (**7**) in tetrahydrofuran;

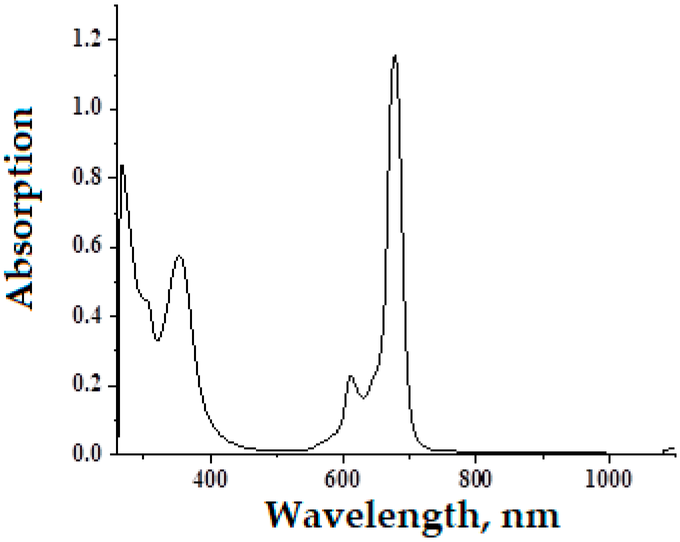

Figure S17. Electronic absorption spectrum of tetrakis-4-[4-(3,4-dicyanophenoxy)phenoxy]-phthalocyaninato ytterbium acetate (**8**) in tetrahydrofuran;

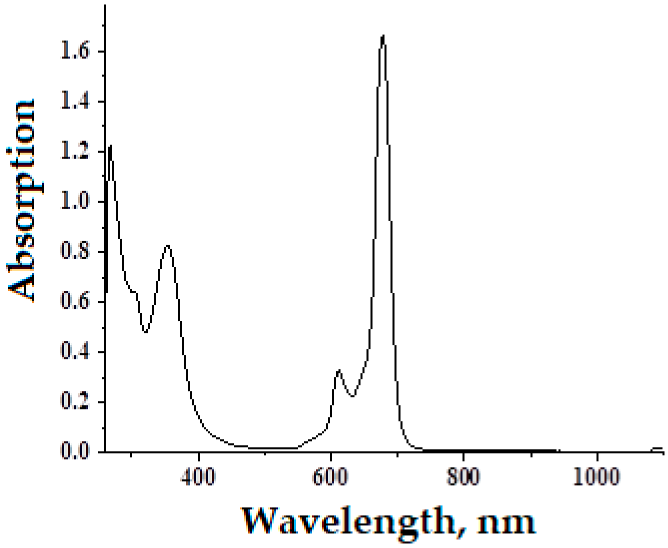

Figure S18. Electronic absorption spectrum of lutetium tetrakis-4-[4-(3,4-dicyanophenoxy)phenoxy]phthalocyaninato acetate (**9**) in tetrahydrofuran.

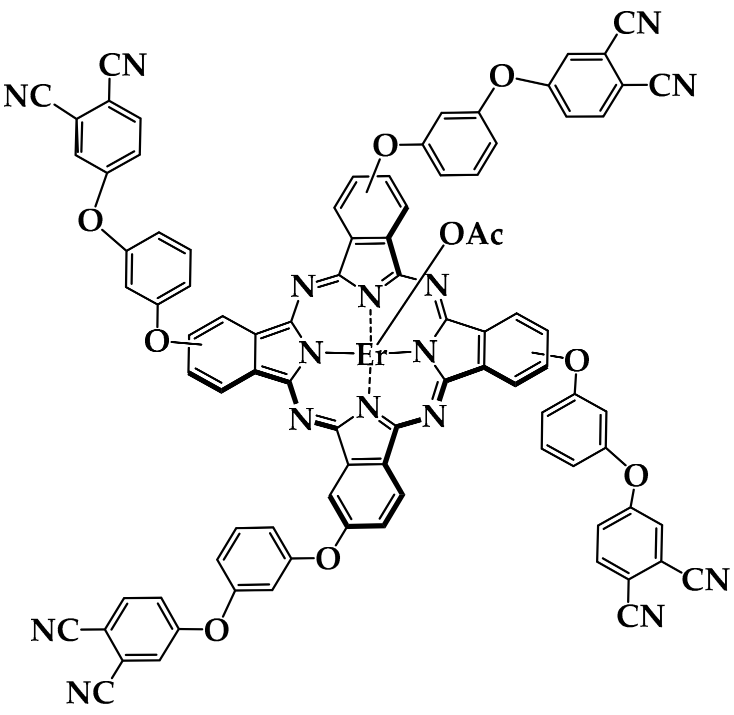

Figure S19. The structural formula of Acetate tetrakis-4-[3-(3,4-dicyanophenoxy)phenoxy]phthalocyaninato erbium (**4**).

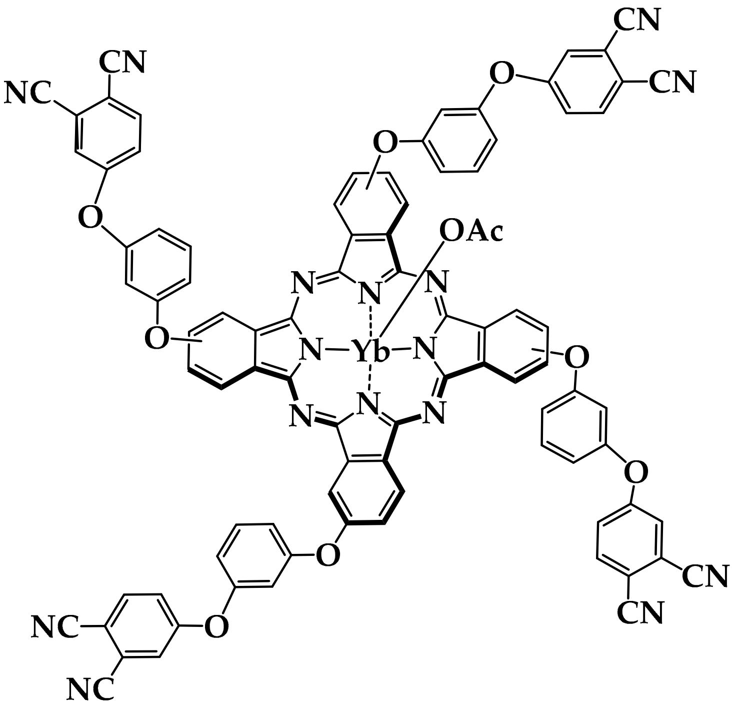

Figure S20. The structural formula of Acetate tetrakis-4-[3-(3,4-dicyanophenoxy)phenoxy]phthalocyaninato ytterbium (**5**).

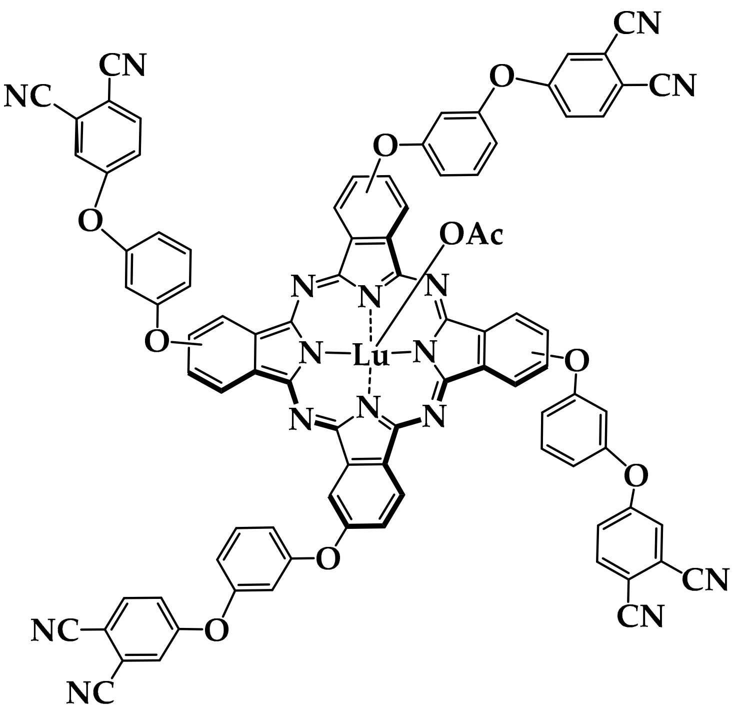

Figure S21. The structural formula of Acetate tetrakis-4-[3-(3,4-dicyanophenoxy)phenoxy]phthalocyaninato lutetium (**6**).

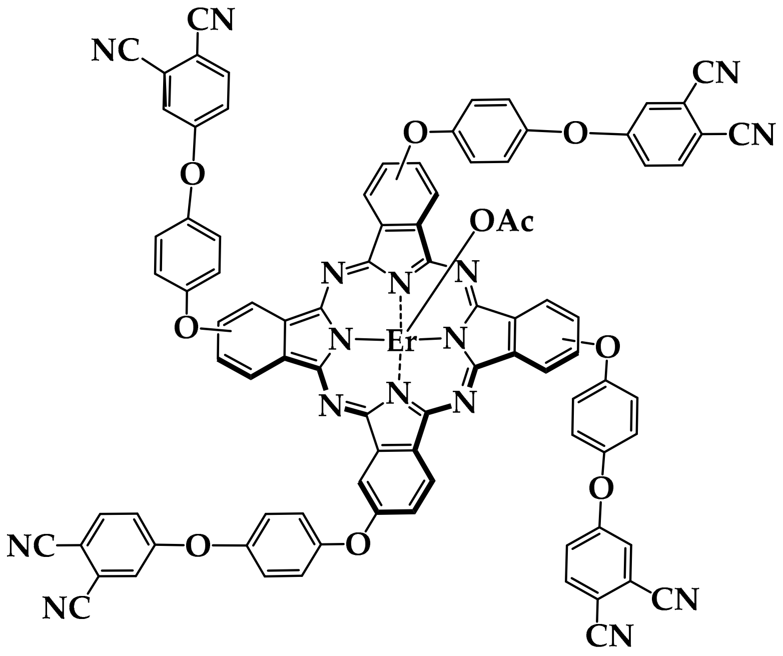

Figure S22. The structural formula of Acetate tetrakis-4-[4-(3,4-dicyanophenoxy)phenoxy]phthalocyaninato erbium (**7**).

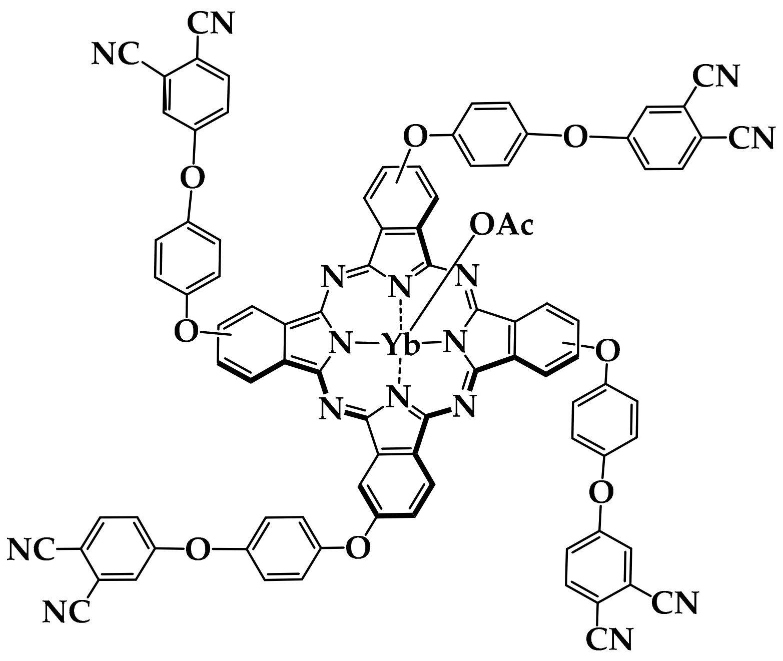

Figure S23. The structural formula of Acetate tetrakis-4-[4-(3,4-dicyanophenoxy)phenoxy]phthalocyaninato ytterbium (**8**).

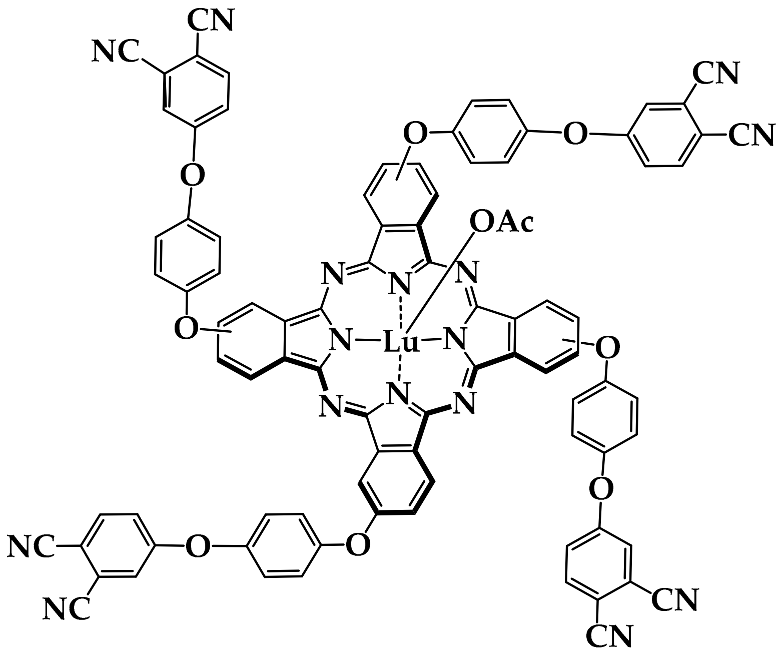

Figure S24. The structural formula of Acetate tetrakis-4-[4-(3,4-dicyanophenoxy)phenoxy]phthalocyaninato lutetium (**9**).

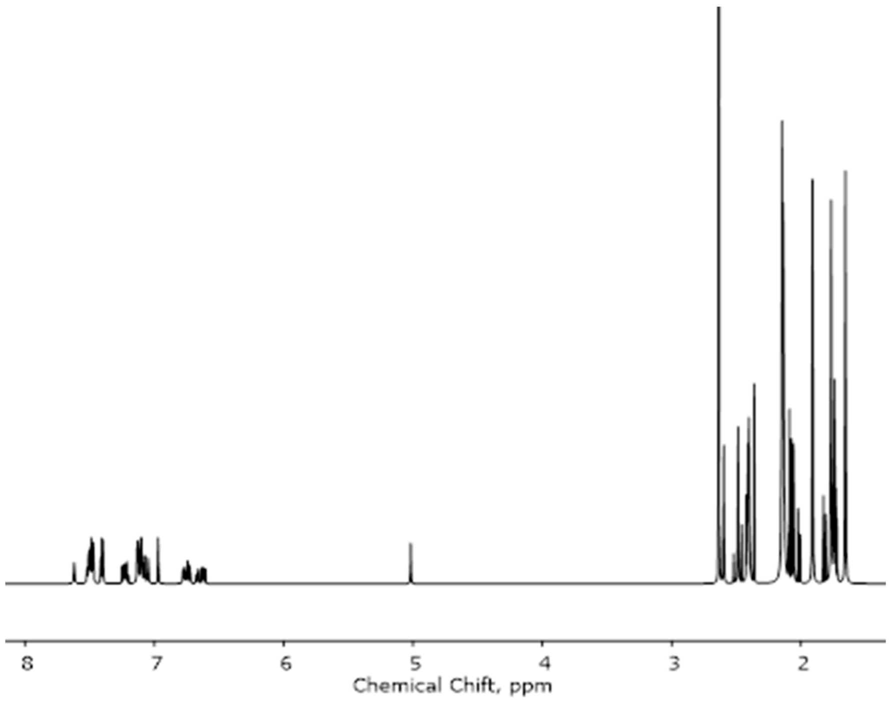

Figure S25. ^{1}H NMR spectrum of Acetate tetrakis-4-[3-(3,4-dicyanophenoxy)phenoxy]phthalocyaninato erbium (**4**) in $CDCl_3$.

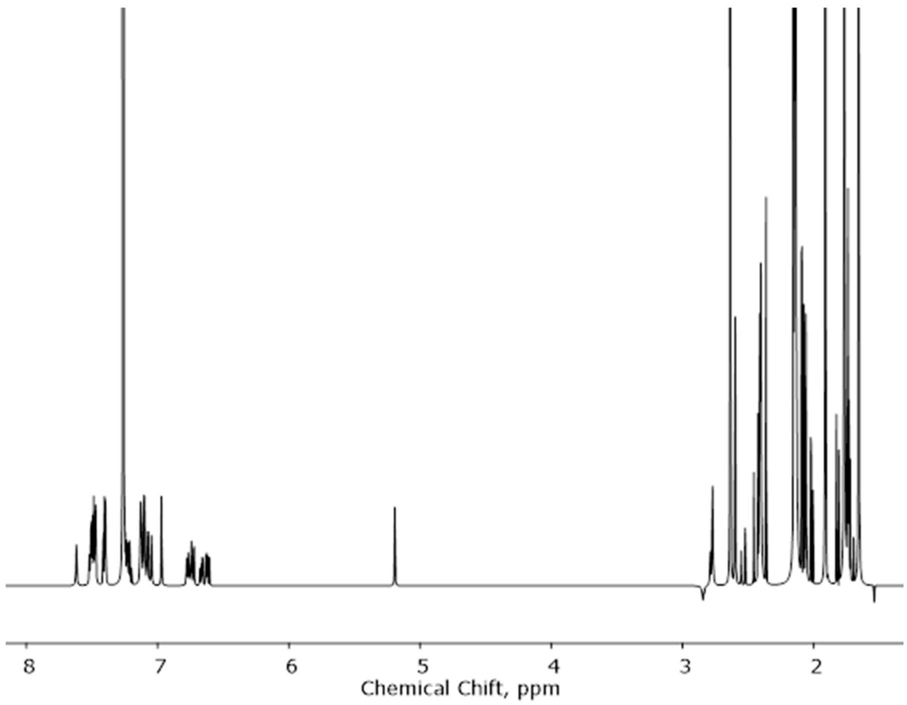

Figure S26. ^{1}H NMR spectrum of Acetate tetrakis-4-[3-(3,4-dicyanophenoxy)phenoxy]phthalocyaninato ytterbium (**5**) in $CDCl_3$.

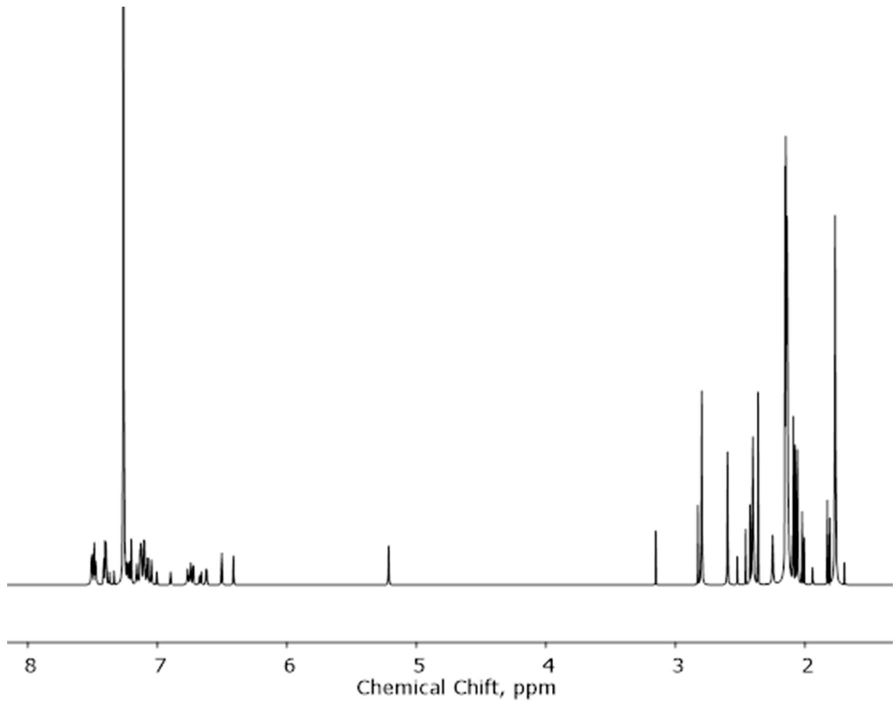

Figure S27. ^{1}H NMR spectrum of Acetate tetrakis-4-[4-(3,4-dicyanophenoxy)phenoxy]phthalocyaninato erbium (**7**) in $CDCl_3$.

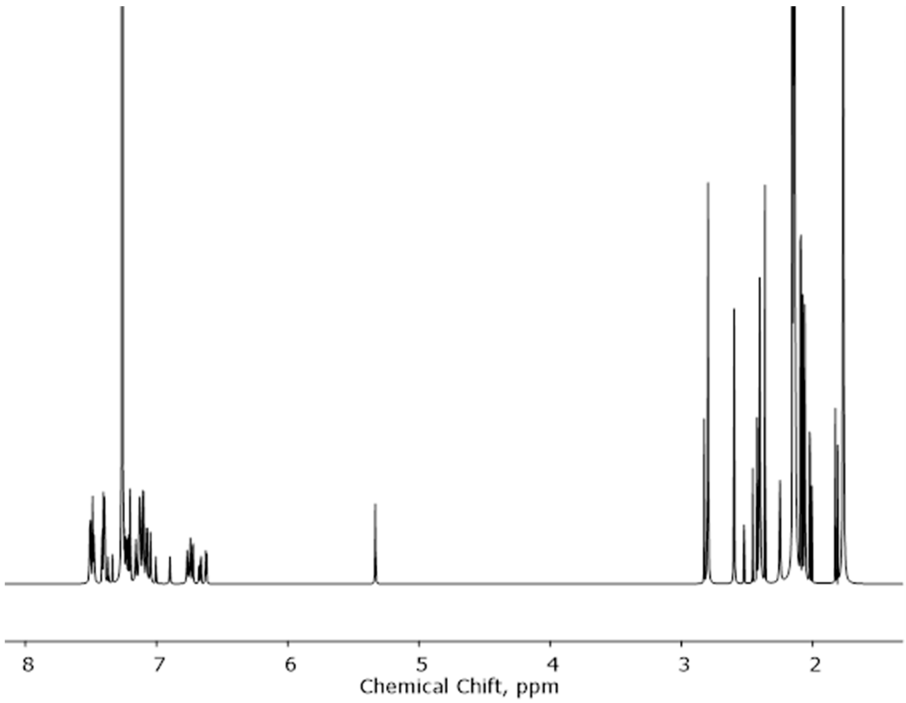

Figure S28. ^{1}H NMR spectrum of Acetate tetrakis-4-[4-(3,4-dicyanophenoxy)phenoxy]phthalocyaninato ytterbium (**8**) in $CDCl_3$.

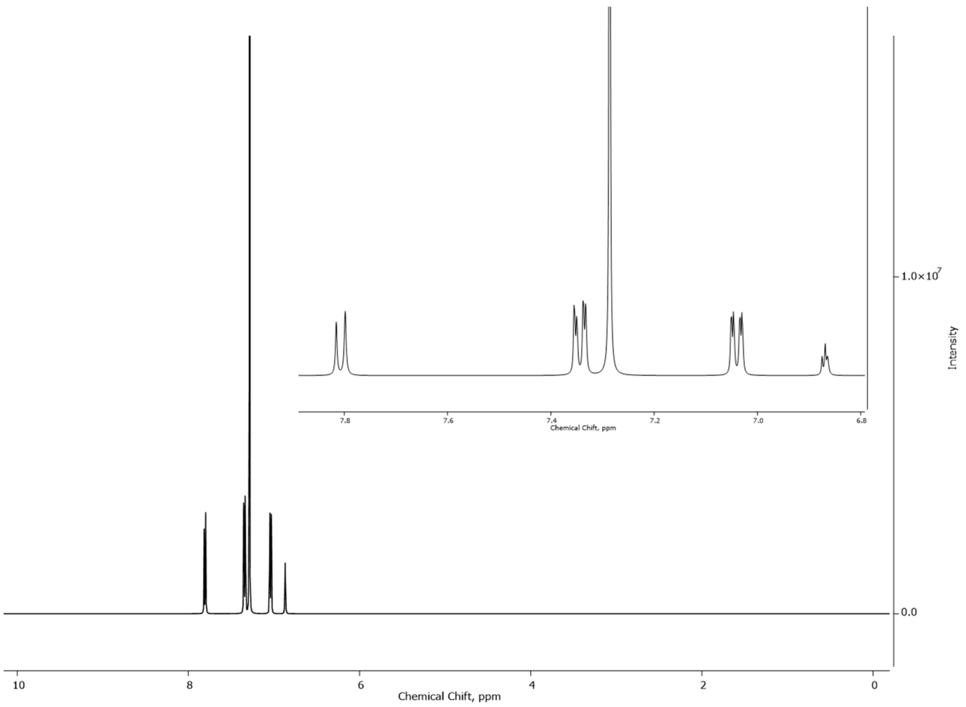

Figure S29. ^{1}H NMR spectrum of Acetate tetrakis-4-[4-(3,4-dicyanophenoxy)phenoxy]phthalocyaninato lutetium (**9**) in $CDCl_3$.

Table S1. FWHT determinations for compounds **4-9** in organic media.

**Compound**	**Δλ, nm (Δλ$_{initial}$ -> Δλ$_{final}$)**		
	**$CHCl_3$**	**Acetone**	**THF**
**4**	26 -> 26	36 -> 27	26 -> 26
**5**	27 -> 27	26 -> 26	26 -> 26
**6**	27 -> 27	26 -> 26	25 -> 25
**7**	26 -> 26	26 -> 26	26 -> 26
**8**	26 -> 26	26 -> 26	25 -> 25
**9**	27 -> 27	35 -> 25	25 -> 25
